# Letter from the Editor in Chief

**DOI:** 10.19102/icrm.2025.16046

**Published:** 2025-04-15

**Authors:** Devi Nair


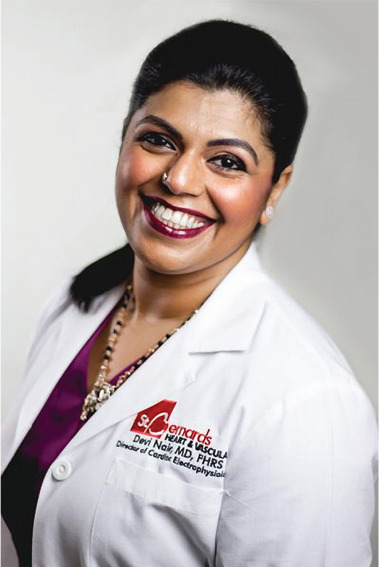


Dear Colleagues,

As we continue to advance the field of cardiac electrophysiology, I am pleased to present the April 2025 issue of *The Journal of Innovations in Cardiac Rhythm Management*. This edition aligns with the recent European Heart Rhythm Association (EHRA) Congress 2025, held from March 30 to April 1 in Vienna, Austria.

## EHRA Congress 2025: A Vienna Symphony of Advances in Arrhythmia Care

This year’s EHRA Congress, themed *“A Vienna Symphony of Advances in Arrhythmia Care,”* convened leading scientists, healthcare professionals, and industry experts to share the latest research and innovations in arrhythmia management. The congress featured more than 120 scientific sessions along with practical tutorials, covering a wide array of topics designed to showcase cutting-edge research and knowledge sharing that are driving the EHRA community forward.

Key highlights from EHRA 2025 include:

- ***Scientific sessions.*** The program included more than 120 sessions focusing on various aspects of arrhythmia care, such as atrial fibrillation, ventricular arrhythmia ablation, and implantable cardioverter-defibrillator therapy.
- ***Practical tutorials.*** Attendees benefited from hands-on courses, including workshops on tracings, intracardiac echocardiography, entrainment, and live heart dissection courses, enhancing practical skills essential for patient care.
- ***Simulation Village.*** The popular Simulation Village provided immersive experiences, allowing participants to engage in realistic scenarios to improve their clinical competencies.
- ***Late-breaking clinical trials.*** Pioneering research was presented in the Late-Breaking Science sessions, offering insights into the latest advancements and their potential impact on clinical practice.

### Commitment to Innovation and Sustainability

EHRA 2025 also highlighted a strong commitment to sustainability and accessibility. The congress aimed to achieve the Austrian Ecolabel certification by implementing eco-friendly practices across mobility, communication, and event management, reflecting a dedication to environmental responsibility.

## Featured Articles in This Issue

In alignment with the themes discussed at EHRA 2025, this issue includes several noteworthy contributions:

- ***“Left Septal Fascicular Block Following Left Bundle Branch Area Pacing.”***This case report by Sertdemir et al. explores the occurrence of left septal fascicular block after left bundle branch area pacing, providing valuable insights into conduction system pacing and its electrocardiographic manifestations.
- ***“Risk Factors Associated with Unsuccessful Dofetilide Initiation Due to Excessive QT Interval Prolongation: A Retrospective Study.”*** This original research by Rast et al. identifies clinical and echocardiographic factors linked to unsuccessful dofetilide initiation, emphasizing the need for careful patient selection to mitigate risks associated with QT interval prolongation.

## Looking Ahead: Heart Rhythm 2025 in San Diego

As we reflect on the insights gained from EHRA 2025, I look forward to the opportunity to continue these discussions in person at the upcoming Heart Rhythm 2025 conference, scheduled for May 14–17, 2025, in San Diego, California. This annual meeting, hosted by the Heart Rhythm Society, serves as a premier forum for sharing advancements and fostering collaborations in cardiac electrophysiology. I encourage all members of our community to attend and contribute to the ongoing evolution of our field.

I extend my gratitude to all contributors, reviewers, and readers for their dedication to advancing the field of cardiac electrophysiology. Together, we continue to strive for excellence in improving patient outcomes and shaping the future of arrhythmia management.

Warm regards,


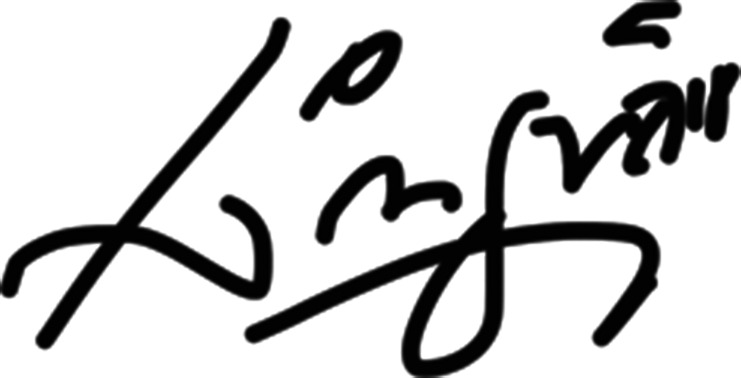


Dr. Devi Nair, md, facc, fhrs

Editor-in-Chief

*The Journal of Innovations in Cardiac Rhythm Management*

Director of the Cardiac Electrophysiology & Research,

St. Bernard’s Heart & Vascular Center, Jonesboro, AR, USA

White River Medical Center, Batesville, AR, USA

President/CEO, Arrhythmia Research Group

Clinical Adjunct Professor, University of Arkansas for Medical Sciences

Governor, Arkansas Chapter of American College of Cardiology

drdgnair@gmail.com

